# Internal Hernia After One Anastomosis Gastric Bypass (OAGB): Lessons Learned from a Retrospective Series of 3368 Consecutive Patients Undergoing OAGB with a Biliopancreatic Limb of 150 cm

**DOI:** 10.1007/s11695-021-05269-1

**Published:** 2021-04-08

**Authors:** Niccolo Petrucciani, Francesco Martini, Radwan Kassir, Gildas Juglard, Celine Hamid, Hubert Boudrie, Olivier Van Haverbeke, Arnaud Liagre

**Affiliations:** 1 Bariatric Surgery Unit, Ramsay Générale de Santé, Clinique des Cedres, Cornebarrieu, France; 2 Department of Medical and Surgical Sciences and Translational Medicine, Faculty of Medicine and Psychology, St Andrea Hospital, Sapienza University, via di Grottarossa 1035-9, 00189 Rome, Italy; 3 Department of Digestive Surgery, CHU Félix Guyon, Saint Denis, La Réunion, France

**Keywords:** Bariatric surgery, One anastomosis gastric bypass, Internal hernia, Complications, Obesity

## Abstract

**Background:**

Internal hernia (IH) represents a relatively common and well-known complication after Roux-en-Y gastric bypass. IH after one anastomosis gastric bypass (OAGB) is less frequent and rarely reported in the literature. This study presents a series of IH after OAGB observed in a high-volume bariatric center.

**Methods:**

Data of patients who underwent OAGB with an afferent limb of 150 cm between May 2010 and September 2019 were prospectively collected and retrospectively analyzed. Data of patients undergoing surgery for IH during follow-up were collected and analyzed.

**Results:**

Ninety-six patients out of 3368 with a history of OAGB had intestinal incarceration in the Petersen’s orifice (2.8%). Specificity and sensitivity of computed tomography scans in the diagnosis of IH were 59% and 76%, respectively. The mean timeframe between OAGB and surgery for IH was 21.9±18.3 months. Mean body mass index at the time of IH surgery was 24.7 ± 3.6. Surgery was completed laparoscopically in 96.8% of cases. Nine patients (9.3%) had signs of bowel hypovascularization. In all patients, the herniated bowel was repositioned, and the Petersen’s orifice was closed, without the need for bowel resection. Mean hospital stay was 1.9 ± 4.8 days. The postoperative morbidity rate was 8.3%. Long-term IH relapse was observed in 14 patients; signs of bowel hypovascularization due to incarceration in a small orifice was observed in eight of these patients (57%).

**Conclusions:**

Incidence of IH after OAGB is 2.8%. IH is associated with a low rate of bowel ischemia and the need for intestinal resection.

## Introduction

Internal hernia (IH) represents a relatively common and potentially serious complication after gastric bypass to treat morbid obesity [1]. After Roux-en-Y gastric bypass (RYGB), IH occurs mainly in the Petersen’s and intermesenteric defect, and its incidence ranges from approximately 1 to 12% in different series [1–5]. Most patients have IH at the intermesenteric defect, whereas IH at the Petersen’s is less common [1]. However, IH at the intermesenteric defect may be very dangerous due to the small size of the orifice. The herniation of the small bowel in mesenteric orifices created by the RYGB procedure may have severe consequences, including small bowel obstruction, ischemia, necrosis, and perforation if the diagnosis and treatment are not prompt [6]. It is mandatory to close the Petersen’s and intermesenteric orifices during RYGB to reduce the incidence of IH. However, the closure of these defects may cause specific complications [7–10]. In mild cases, the IH may also cause chronic digestive symptoms, including pain, discomfort, and nausea [2]. IH is especially dangerous in pregnant women, with reported cases of maternal and fetal death due to bowel ischemia consequent to IH with late diagnosis and treatment [11, 12].

One anastomosis gastric bypass (OAGB) is a relatively recent bariatric procedure, which has rapidly gained acceptance and diffusion worldwide and represents 7.6% of all bariatric interventions. It is the third most frequent procedure following sleeve gastrectomy (SG) and RYGB [13, 14]. Several authors have demonstrated the efficacy and safety of OAGB in treating obesity and its related comorbidities, and the procedure has been recognized by the International Federation for the Surgery of Obesity and Metabolic Disorders (IFSO) as a mainstream bariatric procedure [15–18].

IH occurs less frequently after OAGB than after RYGB. The first case was described in 2016 [19], as a bowel loop incarcerated behind the omega limbs, from the patient’s left to right. IH after OAGB may have different clinical characteristics. However, only a few case reports, video reports, or small series have been published on this subject, therefore, little is known about IH after OAGB.

This study aims to present a series of IH after OAGB observed in a high-volume bariatric center, analyzing the diagnosis, treatment, and outcomes of this complication.

## Patients and Methods

Between May 2010 and September 2019, 3368 patients underwent OAGB with a biliary limb of 150 cm and without closure of the Petersen’s defect. The rationale is that the Petersen’s orifice is usually large after OAGB and bowel strangulation with ischemia is rare. Starting from October 2019, the French National Authority of Health (Haute Autorité de Santé) removed OAGB from the list of reimbursable surgical procedures, therefore, bariatric surgeons in our institution stopped performing OAGB. Data of patients admitted for clinical suspicion of IH between May 2010 and May 2020 were collected and analyzed. Computed tomography (CT) scans with the ingestion of hydrosoluble contrast and injection of intravenous contrast was usually performed for preoperative diagnosis. These patients and patients with the incidental diagnosis of IH during cholecystectomy constitute our study group. Details of patients and disease characteristics were prospectively collected in a computerized database. Patients were retrieved from a prospectively maintained database of all bariatric procedures performed in our institution. The Institutional Review Board approved the present study, which is registered as IORG-IRB: IORG0009085 COS-RGDS-2019-11-001-LIAGRE-A.

### Patient Management and Work-up

All the patients included underwent OAGB with a biliopancreatic limb of 150 cm at our institution, as described previously [20]. The stomach was sectioned at the level of the incisura angularis and calibrated on a 36 Fr bougie to fashion a long and narrow pouch. An antecolic laterolateral gastrojejunal anastomosis was created using a stapler with a 60-mm vascular cartridge. No closure of mesenteric defects was performed. The postoperative follow-up of patients consisted of physical examination and blood tests every 6 months. Patients with acute abdominal pain were admitted to our emergency department and routinely assessed by a bariatric surgeon.

**Fig. 1:**
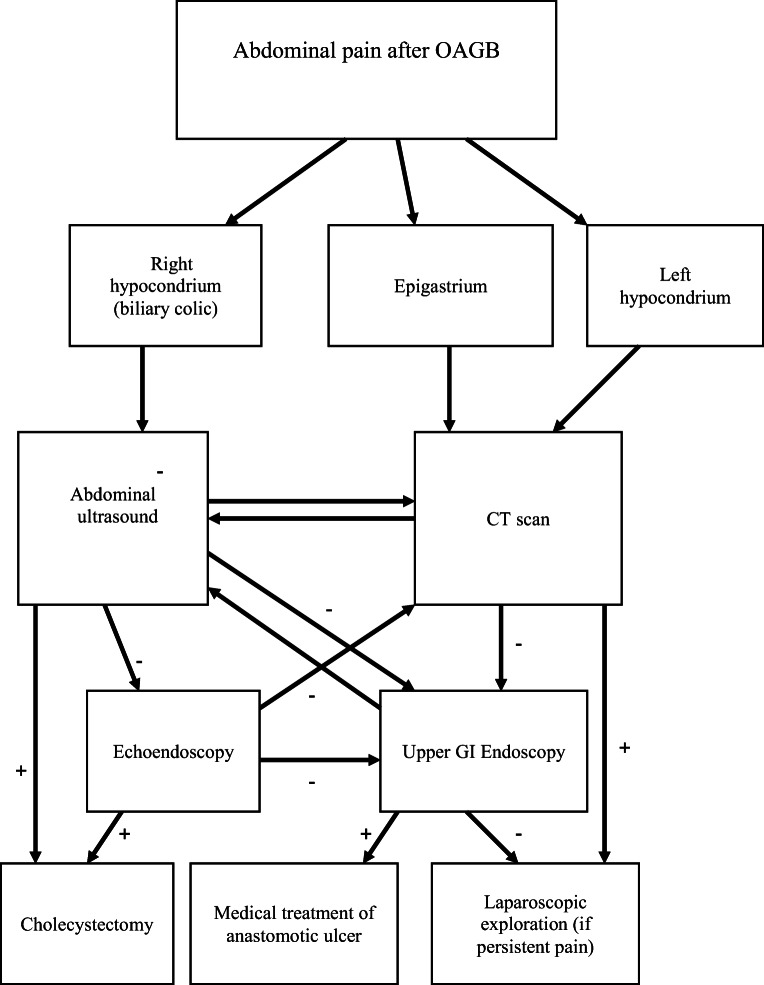
Diagnostic algorithm in patients presenting with abdominal pain after one anastomosis gastric bypass. OAGB, one anastomosis gastric bypass; CT, computed tomography; GI, gastrointestinal

Patients presenting with acute abdominal pain were classified and managed as reported in Fig. 1. Patients presenting with epigastric and left hypochondrial pain underwent CT with oral contrast administration and intravenous contrast injection. If the CT scan was negative, upper GI endoscopy was performed. In cases where anastomotic ulcer was found without perforation, medical treatment was started. If IH was found, the patients underwent laparoscopic exploration. In patients with persistent abdominal pain, laparoscopic exploration was performed according to clinical evaluation.

### Radiological Evaluation (CT Scan with Oral Contrast Administration and Intravenous Contrast Injection)

IH was suspected in case of one or more of these signs: swirl sign, strangulation of the superior mesenteric vein, engorged mesenteric vessels and edema, engorged lymph nodes, ascites, bowel ischemia, or edema, as reported previously [21, 22].

### Surgical Treatment

In cases where there is suspicion of IH and a decision is made for surgical exploration, laparoscopy was performed. The patient was placed with the legs closed and both arms along the body, and the surgeon was at the patient’s right. One trocar was placed in the left hypochondrium to lift the transverse mesocolon and open the Petersen’s space and three more trocars were placed in the right abdomen for the camera and the two hands of the first surgeon. The small bowel was inspected totally starting from the ileocecal valve. The herniated content was repositioned and the mesenteric defect was closed using a running non-resorbable suture (Prolene 2/0).

### Postoperative Outcomes and Follow-up

Postoperative complications were classified according to the Clavien–Dindo classification [23]. Early recurrence of internal hernia was defined as recurrence occurring in the first 30 days after surgery for internal hernia. Patients were examined one month after discharge to assess early postoperative complications. Then, they resumed their normal follow-up rate.

### Statistical Analysis

Data were expressed as mean ± standard deviation or median (range) or as numbers and percentages.

## Results

### Characteristics of Patients

**Fig. 2:**
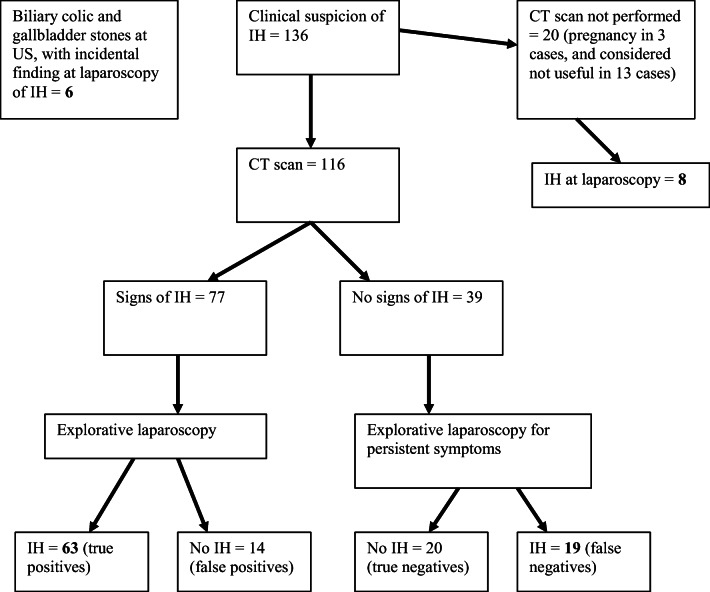
Flowchart of the patients included. US, ultrasonography; CT, computed tomography; IH, internal hernia

Overall, 136 patients were admitted for clinical suspicion of IH. Among these patients, 20 did not undergo a CT scan, three of them because they were pregnant, and 17 because they underwent explorative laparoscopy directly. The latter patients had experienced persistent episodes of pain and already had a negative abdominal ultrasound and GI endoscopy; therefore, a CT scan was not considered useful for diagnostic purposes (Fig. 2).

### Role of CT Scan

In total, 116 patients underwent CT scans; signs of IH were retrieved in 77 patients. IH was confirmed in 63 of these cases, whereas no IH was found in 14 after surgical exploration. No signs of IH were observed in the CT scans of 39 patients but they underwent surgical exploration for persistent symptoms and high clinical suspicion; among them 19 had IH, the remaining 20 had a negative exploration (Fig. 2). The specificity and sensitivity of the CT scans were 59% and 76%, respectively. The positive and negative predictive values of the CT scans were 82% and 51%, respectively. Six other patients had incidental findings and treatment of IH during laparoscopic cholecystectomy for biliary colic.

### Characteristics of Patients Undergoing Surgery for IH

#### Clinical presentation

Ninety-six patients out of 3368 with a history of OAGB had intestinal incarceration in the Petersen’s orifice (2.8%). The mean age was 38.3 ± 11 years (range: 19–71) and 85 patients were female. The location of the abdominal pain was the right hypochondrium, the left hypochondrium, and the epigastrium in 14.5%, 34.5%, and 51.2% of cases, respectively, and it was present for 20.9 ± 33 (range: 1–145) days. Body mass index (BMI) at the time of OAGB was 41.5 ± 4.1 kg/m^2^ (range: 35–56) and weight was 116.2 ± 17.2 kg (range: 88–163). Previous procedures included adjustable gastric banding (AGB) in two cases, SG in two cases, and AGB followed by SG in one case. The mean timeframe between OAGB and surgery for IH was 21.9 ± 18.3 months (range: 3–88). Mean weight and BMI at the time of IH surgery were, respectively, 69.4 ± 12.6 (range: 44–98), and 24.7 ± 3.6 (range: 18–33). Mean kilograms lost after OAGB were 47 ± 12.5 (range: 21–83), and % excess weight loss (EWL) and % total weight loss (TWL) were 104±22 (range: 50–156) and 40±7 (range: 19–58), respectively. An abdominal CT scan with oral contrast administration was performed in 82 patients and was suggestive of IH in 63 (77%). The findings of CT scans are shown in Table 1.

**Table 1.** Findings at CT scan

Finding	*N* (%)
Mesenteric twist ± mesenteric edema	54 (85.8%)
Intestinal invagination	1 (1.6%)
Perianastomotic inflammation	1 (1.6%)
Epiploic inflammation in right hypocondrium	1 (1.6%)
Small bowel obstruction	6 (9.4%)

*CT* computed tomography

#### Surgical Details

Surgery was completed laparoscopically in 93 (96.8%) cases and by laparotomy in three cases for difficult repositioning of the bowel during laparoscopy or because of the low laparoscopic experience of the operating surgeon. Nine patients (9.3%) had signs of bowel hypovascularization in clinical (intense pain), radiological, or peroperative (venous stasis, abdominal fluid) examination. The herniated limb was the common limb in 74 (77%) patients and the biliary limb in 22 (23%). A 180° torsion of the gastrojejunal anastomosis due to quasi-complete incarceration of the common limb was found in 47.3% of patients. The repositioning of the herniated bowel allowed the restoration of the correct positioning of the anastomosis. In all patients, the herniated bowel was repositioned and the Petersen’s orifice was closed, without the need for bowel resections. Additional procedures performed are listed in Table 2.

**Table 2.** Additional procedures during surgery in a series of 96 patients who underwent surgical treatment of internal hernia after one anastomosis gastric bypass

	*N* (%)
Braun anastomosis/conversion to RYGB	1 (1%)
T-tube positioning into a perforated ulcer	1 (1%)
Bowel suture following bowel iatrogenic injury	1 (1%)
Drain positioning	1 (1%)
Umbilical hernia repair	1 (1%)
Cholecystectomy	13 (13.5%)
Bowel resection	0 (0%)

*RYGB* Roux-en-Y gastric bypass

#### Postoperative short and long-term outcomes

Mean hospital stay was 1.9 ± 4.8 days (range: 1–46). The postoperative morbidity rate was 8.3% and mortality was null. Postoperative complications are reported in Table 3.

**Table 3.** Postoperative complications in a series of 96 patients who underwent surgery for an internal hernia after one anastomosis gastric bypass

Complication	Number	Clavien–Dindo grading
Wound infection	1	1
Hypertension	1	2
Pneumonia after inhalation	2	4a
Peritonitis for bowel leak	1	3b
Precocious (within 30 days) relapse of IH	2	3b
Abdominal pain respondent to medical treatment	1	1

*IH* internal hernia

Long-term relapse of IH was observed in 14 patients (14.5%); signs of bowel hypovascularization due to incarceration in a small orifice were found in eight of these patients (57%). Four pregnant women underwent surgery without complications. Three of them were at 12–13 weeks of amenorrhea and underwent direct laparoscopic exploration, one patient at 23 weeks of amenorrhea underwent surgery after a CT scan confirmed a diagnosis of IH. Six patients with a diagnosis of cholecystitis at ultrasound or CT scan underwent laparoscopy, and a concomitant IH was found. In cases with a double diagnosis, it was difficult to determine the etiology of the pain.

## Discussion

IH after OAGB is not a well-known condition. We report in this paper the first series of patients treated for IH after OAGB. In 2016, Facchiano et al. reported the first single case of IH after OAGB [19], and since then only a few case reports have been published about the existence and potential danger of this condition [24–28]. In our institution, OAGB was adopted early and widely and we gathered extensive experience using a modified technique of OAGB with a biliary limb of 150 cm [20, 29, 30]. We could analyze the incidence of IH after OAGB without closure of the mesenteric defects on a series of 3368 patients. The incidence of IH (diagnosed at laparoscopy) was 2.8% (96 patients), which is lower than that reported after RYGB without closure of mesenteric defects [8, 31].

Symptoms of IH may be chronic and not specific, as seen for IH in patients with a history of RYGB. Differential diagnoses include gallbladder diseases, stone migration in the common bile duct, pancreatitis, anastomotic ulcer, gastritis or esophagitis, and small bowel obstruction due to postoperative adhesions. The diagnosis is mainly based on clinical suspicion after a CT scan, with low specificity and sensitivity compared with laparoscopic exploration (59% and 76% in our patients, respectively), and on the exclusion of other possible diagnoses (mainly gallbladder disease and anastomotic ulcer). Laparoscopic exploration was offered also in patients with negative imaging but persistent symptoms suggestive of IH. Six patients with occasional diagnosis at laparoscopy were included. In these patients, the indication for surgery was symptomatic gallbladder lithiasis, but IH was found and repaired, due to the possibility that non-specific symptoms may have been related to bowel passage into the Petersen’s orifice. We recommend systematic exploration of the Petersen’s orifice in patients undergoing laparoscopy for abdominal pain, even if preoperatively another diagnosis was suspected (e.g., biliary colic).

Surgical treatment consists of the closure of the Petersen’s orifice with a non-resorbable suture and is associated with a very low morbidity rate and a short mean hospital stay. Laparoscopy was possible in more than 96% of patients. No cases of bowel ischemia requiring intestinal resection were observed. This is probably related to the large mesenteric defect created by the OAGB, allowing bowel incarceration to strangulate the bowel. In 47.3% of patients, we observed at laparoscopy a 180° torsion of the gastrojejunal anastomosis due to quasi-complete incarceration of the common limb. This observation is peculiar to OAGB: only a single point of fixation exists for the presence of a single anastomosis and the weight of the herniated bowel may easily cause rotation of the anastomosis. This phenomenon is never observed in the case of IH after RYGB, where the two anastomoses represent two points of fixation. It should be highlighted that the authors experienced a higher risk of bowel ischemia in patients with recurrent IH after OAGB. In those patients, the Petersen’s orifice was only partially open and so it was smaller, leading to a higher rate of bowel ischemia, which was present in eight out of 14 patients. In our experience, more than one recurrence may be possible in the same patient during time.

No clear recommendations exist on the closure of Petersen’s orifice during OAGB. This study identifies the rate of secondary surgery to treat an IH in a large series of patients, and the associated short and long-term outcomes. According to our experience, IH after OAGB is rarely associated with serious complications such as bowel ischemia.

We highlight several important points. The organization and timing of patients’ treatment are very important to avoid wasting time and to reduce the incidence of bowel necrosis. Our patients are instructed about potentially dangerous symptoms and are encouraged to directly consult the bariatric surgical unit, which guarantees rapid and expert management. The referral of patients to centers lacking experience in bariatric surgery may be responsible for diagnostic and treatment delay [11]. Furthermore, in our opinion, the experience of the center and the number of bariatric surgeries per year is a second important factor. We believe that this is one of the factors permitting no cases of bowel necrosis out of 3368 OAGB in our institution.

The advantage of closure of the Petersen’s space is the theoretical diminution of the incidence of IH (which however has not been proven after OAGB, whereas it has been demonstrated after RYGB). Disadvantages of the closure are (1) the creation of a smaller space in case of incomplete or failing of the closure, which may results in IH with higher rate of bowel strangulation; (2) the increased operative time; and (3) potential complications related to this suture, such as mesenteric hematomas and bowel kinking.

Even if mesenteric defect closure is recommended during RYGB, we decided not to close the Petersen’s defect during OAGB. The rate of ischemia of the herniated bowel was low (9.3%), and no resections were needed. However, patients with relapsing IH had smaller orifices and a higher rate of bowel ischemia (57%). Our data are not in favor of a systematic closure of Petersen’s defect during OAGB**.** We do not recommend systematic closure of the Petersen’s space, in the lack of robust evidence on this topic. We highlight that this represents the authors’ opinion based only on their personal experience. Further studies, ideally randomized studies about IH after OAGB, comparing closure versus non-closure, should be encouraged to provide high-grade evidence.

### Limits

The present study is limited by its single-center and retrospective design. However, several strengths of the study should be highlighted: the remarkable number of enrolled patients (considering the rarity of the disease), and the new information on this disease that is provided.

## Conclusion

The incidence of IH treated by surgical repair was 2.8% after OAGB without closure of the Petersen’s defect, in a series including a total of 3368 patients. The rate of intestinal resection was null, the rate of bowel reversible ischemia was low, and patients had a low rate of postoperative complications and a short hospital stay.
